# Exploring the Complex Network of Heme-Triggered Effects on the Blood Coagulation System

**DOI:** 10.3390/jcm11195975

**Published:** 2022-10-10

**Authors:** Sarah Mubeen, Daniel Domingo-Fernández, Sara Díaz del Ser, Dhwani M. Solanki, Alpha T. Kodamullil, Martin Hofmann-Apitius, Marie-T. Hopp, Diana Imhof

**Affiliations:** 1 Department of Bioinformatics, Fraunhofer Institute for Algorithms and Scientific Computing (SCAI), Schloss Birlinghoven, D-53757 Sankt Augustin, Germany; 2 Enveda Biosciences, Inc., San Francisco, CA 94080, USA; 3 Polytechnic University of Madrid, E-28040 Madrid, Spain; 4 Pharmaceutical Biochemistry and Bioanalytics, Pharmaceutical Institute, University of Bonn, An der Immenburg 4, D-53121 Bonn, Germany; 5 Causality Biomodels, Kinfra Hi-Tech Park, Kalamassery, Cochin 683503, Kerala, India

**Keywords:** blood coagulation cascade, data mining, heme, hemolysis, knowledge graph, platelet activation, thrombosis

## Abstract

Excess labile heme, occurring under hemolytic conditions, displays a versatile modulator in the blood coagulation system. As such, heme provokes prothrombotic states, either by binding to plasma proteins or through interaction with participating cell types. However, despite several independent reports on these effects, apparently contradictory observations and significant knowledge gaps characterize this relationship, which hampers a complete understanding of heme-driven coagulopathies and the development of suitable and specific treatment options. Thus, the computational exploration of the complex network of heme-triggered effects in the blood coagulation system is presented herein. Combining hemostasis- and heme-specific terminology, the knowledge available thus far was curated and modeled in a mechanistic interactome. Further, these data were incorporated in the earlier established heme knowledge graph, “HemeKG”, to better comprehend the knowledge surrounding heme biology. Finally, a pathway enrichment analysis of these data provided deep insights into so far unknown links and novel experimental targets within the blood coagulation cascade and platelet activation pathways for further investigation of the prothrombotic nature of heme. In summary, this study allows, for the first time, a detailed network analysis of the effects of heme in the blood coagulation system.

## 1. Introduction

Hemolysis-associated thrombosis is a common complication observed in diseases, such as sickle cell disease (SCD) and paroxysmal nocturnal hemoglobinuria (PNH), or as a side effect of transfusions [1,2,3]. The cumulative incidence for thrombosis with ~11–27% in autoimmune hemolytic anemia, ~17% in SCD, and ~29–44% in PNH is as high as in inherited thrombophilias, such as protein C deficiency (~21%) [4,5,6]. Thereby, predominantly venous thrombotic events occur, encompassing deep vein thrombosis and pulmonary embolism, which can even lead to death in the most severe cases [4,6,7]. Among other disorder-specific factors, such as glycosylphosphatidylinositol anchor deficiency in PNH or vessel obstruction by sickle-shaped red blood cells in SCD, the major pathophysiological event underlying these hypercoagulopathies is intravascular hemolysis, which is characterized by an excessive, premature rupture of red blood cells that ultimately leads to a massive release and accumulation of hemoglobin and heme into the bloodstream [8,9]. Both are rapidly scavenged and cleared by the respective plasma proteins, involving haptoglobin, albumin, and hemopexin [10,11,12,13,14]. However, overwhelming of the heme-binding capacity of the plasma (~1.2–1.8 mM [15,16]) provokes the accumulation of labile heme. Labile heme, in turn, has been shown to be capable of binding and functionally affecting a wide range of plasma proteins, thereby causing most of the observed clinical outcomes in hemolytic disorders [9]. The underlying signaling pathways were recently contextualized in the Heme Knowledge Graph (HemeKG), which provided novel mechanistic insights at the molecular level [17]. Specifically, Toll-like receptor 4 (TLR4) signaling was identified as the main route for heme-driven proinflammatory action [17,18,19]. The exact mechanism of the pathway activation has not yet been unraveled; however, evidence for heme-mediated TLR4 activation has been associated with increased excretion of proinflammatory cytokines, elevated complement deposits on endothelial cells, and vasoocclusion [18,20,21]. Furthermore, the main driver of the complement system, component 3 (C3), binds, among other proteins, heme, which results in the deposition of activation fragments on endothelial cells and, thus, complement overactivation, as has been monitored in hemolytic diseases [21,22,23,24,25]. The versatile effects of heme as a modulator in the blood coagulation system have also been extensively described, -either as a matter of side effects during heme injection for the treatment of acute intermittent porphyria (AIP) or because of excessive heme release in hemolytic disorders, such as SCD and PNH [4,26,27]. Thus, initial efforts were made to investigate the molecular basis of the interference of heme in the coagulation system, identifying a few proteins that are affected by heme [26]. However, the currently available data are partially contradictory with respect to the pathophysiological outcome (bleeding vs. thrombosis) and exhibit conspicuous knowledge gaps with respect to the broad spectrum and complexity of the blood coagulation system with its primary (platelet adhesion, activation, and aggregation) and secondary (enzymatic clotting cascade) pathways.

The present study aimed at mapping and contextualizing the current knowledge on heme-driven thrombosis. We therefore established a novel knowledge graph, called “HemeThrombKG”, focusing on heme-driven effects in the blood coagulation system. A pathway analysis, which includes information from three pathway databases, sheds light on important effector proteins that were not analyzed in the context of heme so far, revealing future targets for further exploration of the molecular basis of heme-driven thrombosis. Finally, the extension of the earlier established HemeKG by the novel heme-thrombosis knowledge graph provides a large network on heme-driven effects under hemolytic conditions, comprising a total of more than 800 nodes and 3000 relations, which is freely accessible and will enable researchers and physicians to independently explore the heme biology network for future study development.

## 2. Materials and Methods

### 2.1. Knowledge Modeling and Inclusion of the Knowledge Graph into HemeKG

**Figure 1:**
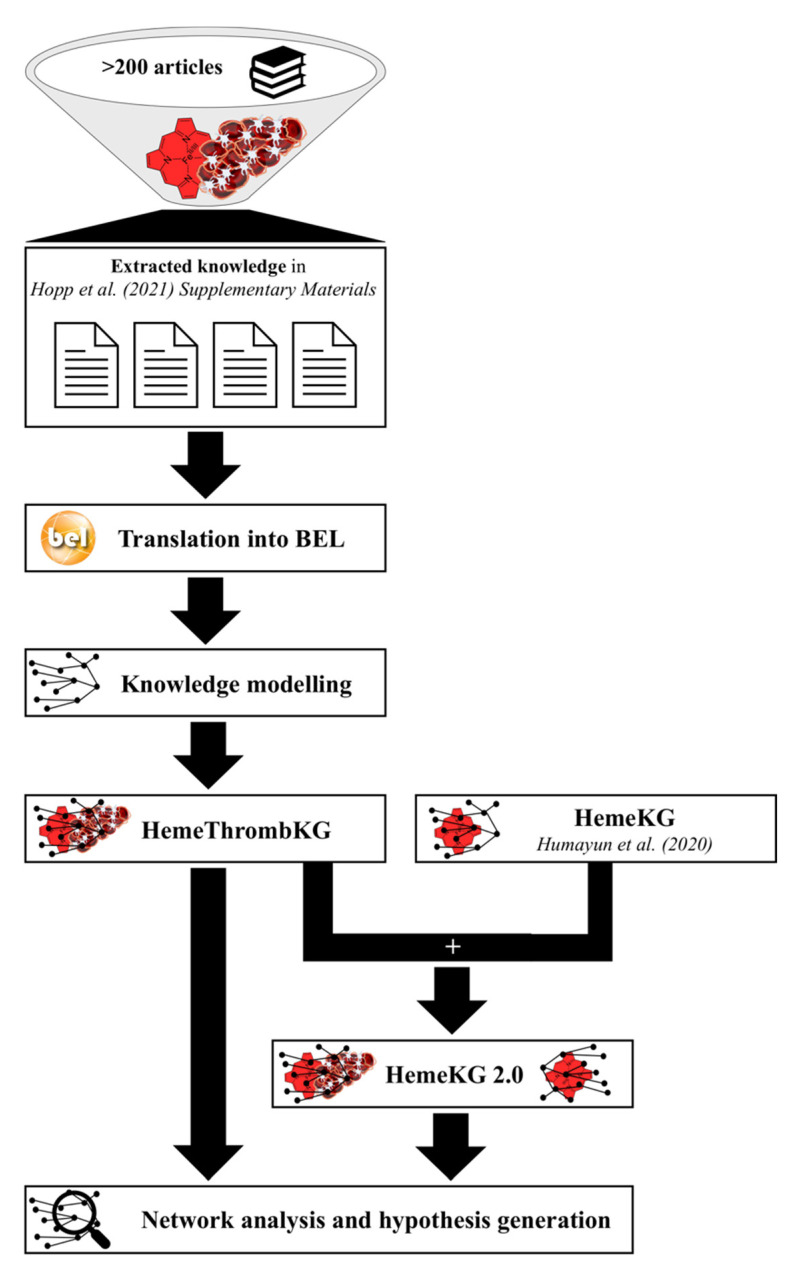
Workflow for the generation, progression, and analysis of HemeThrombKG. Using the knowledge comprehensively collected in a recent review article by the authors [26], the current information about heme’s interference in the blood coagulation system was extracted and translated into BEL to generate the novel knowledge graph “HemeThrombKG”. Furthermore, this new network of heme effects was included in the earlier established knowledge graph “HemeKG” [17], resulting in the expanded knowledge graph on heme biology “HemeKG 2.0”. Both computational networks can be used for pathway and causality analysis, which can be applied for e.g., basic research on the effects of heme but also for the development of suitable drugs for the treatment of hemolysis-driven pathophysiology, such as thrombosis.

An earlier published review article concerning the link between heme and thrombosis [26], which presented knowledge from over 200 articles, formed the basis for the present study. Therein, knowledge on (1) the side effects observed after heme injection, (2) the impact of heme intoxication on cells participating in blood coagulation, and (3) the effect of heme on proteins acting in blood coagulation is depicted in three supplementary tables [26]. This information was used to construct a knowledge graph, specifically focusing on the relations between heme and the development of coagulation disorders. As previously described [17], the detailed knowledge was extracted and manually coded into biological expression language (BEL; Figure 1), incorporating information about the experimental setting (e.g., dose of heme, type of injection, and cell type) as well as binding affinity data if applicable. While “heme”, “hemin”, and “hematin” were consistently curated as “heme”, formulations of heme (e.g., heme arginate and heme-albumin formulations) were excluded, since these were established to prevent from heme-driven side effects in the treatment of porphyrias (heme deficiency diseases). The BEL statements were then used to generate a knowledge graph, designated as “HemeThrombKG” in the following, which models the different effects caused by heme in the context of thrombosis and/or bleeding and thus, was subjected to further analysis. Furthermore, we enriched the earlier established HemeKG with these coagulation-related data by merging the HemeKG with the novel HemeThrombKG (Figure 1).

### 2.2. Analysis of the Crosstalk of Heme with the Blood Coagulation System

First, the relevant pathways for blood coagulation at the molecular level were selected and extracted from three pathway databases KEGG [28], Reactome [29], and WikiPathways [30], comprising the platelet activation pathway (KEGG, HSA04611), the extrinsic pathway of fibrin clot formation (Reactome, R-HSA-140834), the intrinsic pathway of fibrin clot formation (Reactome, R-HSA-140837), the common pathway of fibrin clot formation (Reactome, R-HSA-140875), the platelet aggregation/plug formation (Reactome, R-HSA-76009), and the blood clotting cascade (WikiPathways, WP272).

The data from these pathways were combined and explored by using the web application PathMe [31], resulting in a network with more than 400 edges (Figure S1). Subsequently, this network was used to enrich the generated HemeThrombKG by integrating this knowledge into the original knowledge graph, as described earlier [17,32]. The enrichment led to a network consisting of 848 nodes (including 246 proteins) and 3430 edges. Due to the size of the graph, the investigation of common components was split into the analysis of common extracellular plasma proteins and effects on the cellular level with respect to common membrane-associated and intracellular proteins. For that purpose, the list of 246 proteins was screened for their location (extracellular vs. intracellular and transmembrane proteins) using annotations available from QuickGO [33]. Subsequently, the extracellular and the intracellular/membrane-associated proteins from the database network were independently overlaid with the protein network extracted from HemeThrombKG. In total, 22 extracellular proteins, 10 transmembrane and membrane-associated proteins (incl. adhesion proteins, channels, and receptors), and 18 intracellular proteins were found in the HemeThrombKG, whereas the network extracted from the databases encompassed 54 extracellular proteins, 24 transmembrane and membrane-associated proteins, and 101 intracellular proteins. For clarity, the analysis of the extracellular pathway (i.e., the blood coagulation cascade) was split into the intrinsic, the extrinsic, and the common pathway, as provided by Reactome (R-HSA-140834, R-HSA-140837, and R-HSA-140875 [29]). In the case of membrane-associated proteins and intracellular proteins, a pathway-level analysis was performed by using HemeKG 2.0 in order to include a greater number of relevant proteins. Due to the highest overlap, the pathway-level analysis was performed by focusing on the platelet activation signaling pathways as provided by KEGG (HSA04611 [28]). Common nodes were highlighted, and the node size automatically arranged according to the abundance in the underlying BEL relations.

## 3. Results

### 3.1. HemeThrombKG Illustrates the Knowledge about Heme’s Interference in the Blood Coagulation System

**Figure 2:**
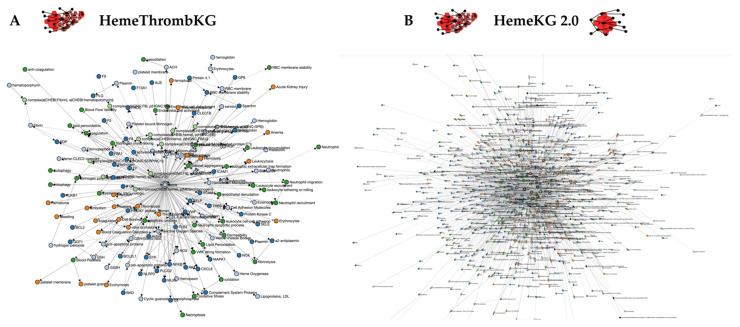
(**A**) The novel HemeThrombKG, consisting of 151 nodes and 426 edges, comprises the current knowledge of heme’s interferences in the blood coagulation system. (**B**) HemeKG 2.0 displays the extended knowledge graph of the earlier established HemeKG [17] by inclusion of the relations of HemeThrombKG. The network combines 868 nodes and 3430 edges. Nodes are colored according to their different functions in BEL (blue: protein, orange: pathology, green: biological process, red: miRNA, light green: complex, black: reaction, light orange: gene, light blue: abundance, pink: RNA).

After the establishment of the first knowledge graph on heme biology (“HemeKG”) in 2020 [17], in which data regarding heme-driven thrombosis were underrepresented, the herein introduced knowledge graph “HemeThrombKG” specifically focuses on the effects of heme on the blood coagulation system. HemeThrombKG is based on extracted and filtered knowledge from over 200 publications over the last 110 years, which was comprehensively combined in a recent review [26]. HemeThrombKG contains 151 nodes and 426 edges with 47 proteins involved (Figure 2A). In addition, contextual information, such as cell type and heme-binding kinetics, is deposited where applicable. In particular, lodging of the heme-binding affinity data will allow to rank the proteins for mechanistic analysis of heme-driven coagulation disorders in the future. Since administered heme for the treatment of AIP was reported to cause prothrombotic effects (e.g., thrombophlebitis), clinical symptoms that occur upon heme injection were incorporated in the network as well.

To further extend and network the knowledge contained in the earlier established HemeKG [17], the relations from HemeThrombKG were incorporated into HemeKG. In the following, the combined knowledge graph is designated as “HemeKG 2.0” and consists of 868 nodes (incl. 246 proteins) and 3430 edges (Figure 2B).

Finally, to allow researchers global access to the network, the BEL documents of the curated content have been made publicly available (https://github.com/HemeThrombKG/HemeThrombKG).

### 3.2. HemeThrombKG and HemeKG 2.0 Enable the Detailed Analysis of Heme-Triggered Thrombosis at the Molecular Level

**Figure 3:**
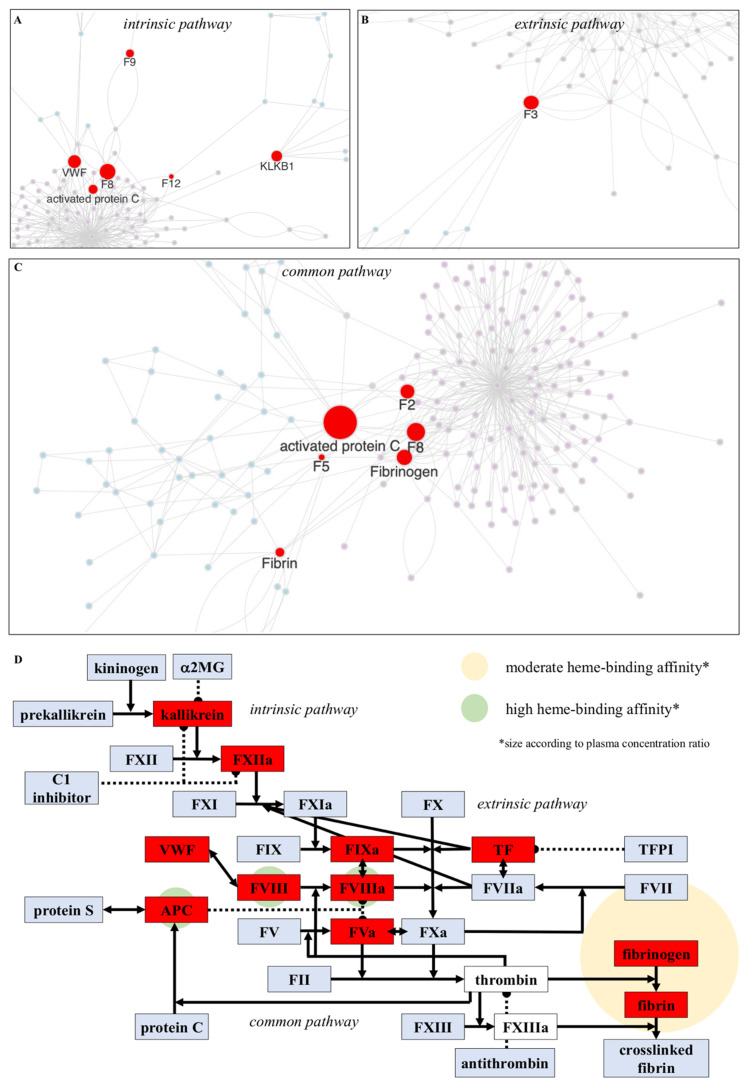
Crosstalk analysis of the blood coagulation cascade and HemeThrombKG. (**A**) The overlay of HemeThrombKG (light purple) and the intrinsic pathway (light blue) of the blood coagulation cascade (Reactome, R-HSA-140837) revealed plasma kallikrein, the coagulation factors VIII, IX, and XII, as well as APC and VWF as common nodes. (**B**) When superimposed with the extrinsic pathway (Reactome, R-HSA-140834; light blue), only TF appeared as a protein affected by heme. (**C**) In the common pathway (light blue), crosstalk analysis demonstrated thrombin, APC, coagulation factors V and VIII, as well as fibrinogen and fibrin as common proteins that are affected in the usual coagulation process as well as in heme-triggered thrombosis. (**D**) The superimposition of the complete blood coagulation cascade (Reactome, R-HSA-140877) with HemeThrombKG demonstrated that heme is capable of affecting the intrinsic, the extrinsic, and the common pathway of the cascade. Common effector proteins as found in the crosstalk analysis are highlighted (dark red). Recently, it was shown that the enzymatic function of thrombin and FXIIIa is not influenced by heme (white). Direct heme binding was only demonstrated for APC, FVIII/FVIIIa, and fibrinogen (circles depict the relation of their plasma concentration and heme-binding affinity; see legend in the figure on the right). Further search for a correlation of the remaining participating proteins and heme in the literature did not reveal any further relations, which highlights these effector proteins as interesting future targets. F2: thrombin, F3: tissue factor, F5: coagulation factor Va, F8: coagulation factor VIII(a), F9: coagulation factor IXa, F12: coagulation factor XIIa, KLKB1: plasma kallikrein.

**Figure 4:**
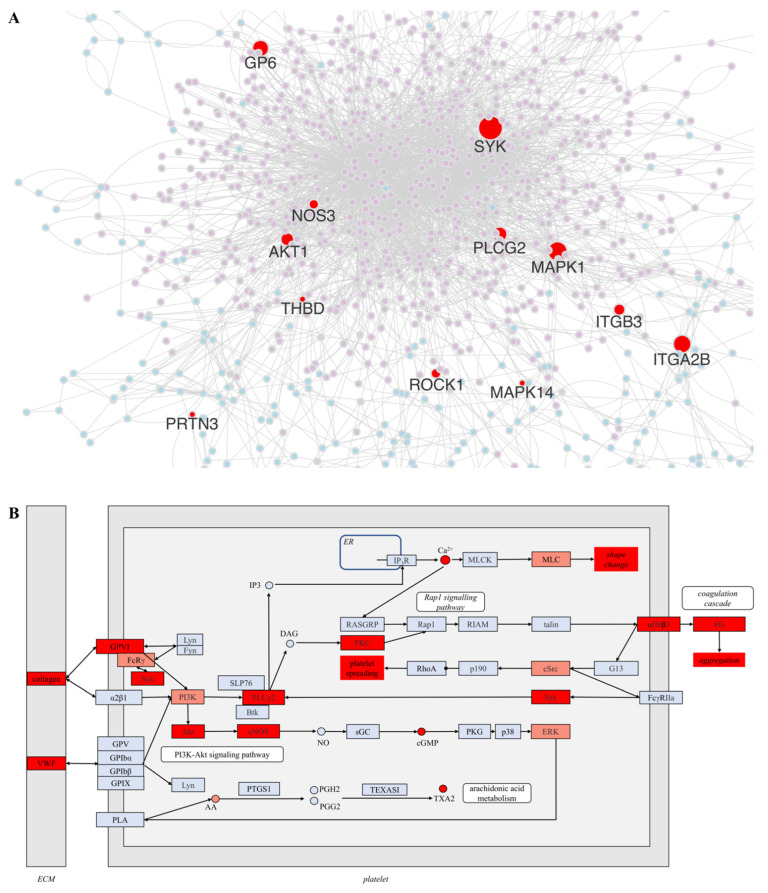
Heme-affected membrane-associated and intracellular proteins in clotting processes. (**A**) The overlay of HemeThrombKG (light purple nodes) and the clotting pathways extracted from databases (light blue nodes) reveals several membrane-associated (i.e., receptors and integrins) and intracellular proteins as common nodes (red). (**B**) HemeKG 2.0 shares distinct proteins with the platelet activation signaling pathways (HSA04611, KEGG [28]). The pathways with the highest level of overlap are depicted, with the common proteins highlighted in red. Relevant small molecules, i.e., IP3, DAG, PGH2, PGG2, TXA2, NO, and cGMP, Ca^2+^ ions, as well as essential platelet activation processes (e.g., spreading, aggregation) are included. Effector proteins that are not included in HemeKG 2.0 but reported in the context of heme signaling are marked in light red, whereas molecules that were not yet described in relation with heme are shown in light blue. AKT: AKT serine/threonine kinase, Btk: Bruton’s tyrosine kinase, DAG: diacylglycerol, ECM: extracellular matrix, ER: endoplasmatic reticulum, ERK: extracellular-signal regulated kinase, FCγRIIa: FCγ receptor IIa, FcRγ: Fc receptor γ, FG: fibrinogen, GPV/Ibα/Ibβ/IX: glycoproteins V/Ibα/Ibβ/IX, GP6/GPVI: platelet glycoprotein VI, IP3: inositol trisphosphate, IP3R: inositol trisphosphate receptor, ITGA2B + ITGB3: integrin αIIbβ3, MAPK: mitogen-activated protein kinase, MLC: myosin light chain, MLCK: myosin light chain kinase, NOS: nitric oxide synthase, PI3K: phosphoinositide 3 kinase, PIP2: phosphatidylinositol-4,5-bisphosphate, PKC: protein kinase C, PKG: cGMP-dependent protein kinase or protein kinase G, PLA: phospholipase A, PLCG2/PLCγ2: phospholipase C γ2, PRTN3: proteinase 3, PTGS1: cyclooxygenase 1, RASGRP: RAS guanyl releasing protein 1, RhoA: Ras homolog family member A, RIAM: amyloid beta precursor protein binding family B member 1 interacting protein, ROCK1: Rho kinase 1, sGC: soluble guanylate cyclase, SLP76: SH2 domain-containing leukocyte protein of 76 kDa; SYK: spleen-associated tyrosine kinase, TEXAS1: thromboxane A synthase, VWF: von Willebrand factor.

As described earlier [17,34], a knowledge graph is highly conducive to study the crosstalk of the effects that are driven by heme under hemolytic conditions with pathways or pathophysiologies of interest. To explore the pathway crosstalk of heme with the underlying pathways of the blood coagulation system, HemeThrombKG was enriched with the relevant pathways from the three databases KEGG [28], Reactome [29], and WikiPathways [30], which showed the greatest overlap with the process of fibrin clot formation (blood coagulation cascade) with respect to plasma proteins and platelet activation signaling in the case of membrane-associated and intracellular proteins (Figure 3 and Figure 4). Thus, the crosstalk analysis focused on these pathways, which is described in detail in the following subsections.

#### 3.2.1. Crosstalk of Heme and the Plasma Proteins of the Blood Coagulation System

To study the crosstalk of heme-driven signaling with the blood coagulation cascade, the network of HemeThrombKG was separately superimposed with each of the three parts (i.e., intrinsic, extrinsic, and common pathway) of the cascade (Figure 3A–C). The blood coagulation cascade can be initiated by the activation of the coagulation factor XII (intrinsic pathway) on the one hand and cellular exposure of tissue factor to the bloodstream (extrinsic pathway) on the other hand. Both pathways culminate in the common pathway, which finally leads to the formation of a fibrin clot.

Superimposition of the HemeThrombKG network and the intrinsic pathway (Reactome, R-HSA-140837) revealed the coagulation factors VIII, IX, and XII, the coagulation inhibitor activated protein C (APC), the adhesive protein von Willebrand factor (VWF), and plasma kallikrein as common nodes, which were described in the past to bind and/or to be affected by heme in vitro and partially in vivo [20,35,36,37,38,39] (Figure 3A).

One of the initial intrinsic pathway serine proteases, plasma kallikrein is activated in the presence of heme (up to 24 nmol), leading to the procoagulant induction of the intrinsic pathway in plasma samples [35]. In the same context, heme-triggered autoactivation of FXII has been suggested [35]. However, upon retroorbital injection (up to 35 µmol/kg) into mice, immunoblockage of FXII did not reduce the heme-triggered coagulation activation, which is why a role of the proposed FXII-heme interaction in vivo appears questionable [40].

Apart from potential procoagulant effects on these proteins of the intrinsic pathway, high-affinity heme binding [K_D_ ~1.9 nM (FVIII) and K_D_ ~12.7 nM (FVIIIa)] to FVIII(a) abolishes its interaction with FIX, which ultimately leads to the inhibition of the clotting process in vitro [37]. In contrast, persistent FVIII-VWF complex formation and increased binding of this complex to human platelets in the presence of heme directs again towards procoagulant signaling [38]. VWF itself has been found to show higher expression levels, string formation, and secretion from Weibel–Palade bodies upon incubation of endothelial cells with heme (up to 100 µM; in vitro) and/or heme injection (3.2 µmol/kg) into mice (in vivo) [20,23]. In vitro, VWF was also capable of the protection of FVIII(a) from its heme-driven inhibition [37]. Furthermore, heme upregulates proteases (e.g., MMP9), which regulate VWF digestion, as has been observed in plasma from SCD patients and with heme (up to 60 µM) incubated endothelial cells [39]. In line with these procoagulant effects on FVIII and VWF, the function of the natural inactivator of FVIIIa, APC, is completely abrogated upon direct heme binding (K_D_ ~400 nM) to the serine protease in vitro (up to 100 µM heme), tending towards prothrombotic consequences of heme excess as well [36].

The superimposition of HemeThrombKG with the extrinsic clotting pathway (Reactome, R-HSA-140834) revealed tissue factor (TF) as a common component and, thus, as the only so far known heme-affected factor in the extrinsic pathway (Figure 3B). Thereby, heme initiates the extrinsic pathway by induction of TF expression and thus provision of an increased level of functionally active TF [40,41,42]. This was observed in different cell types (i.e., endothelial cells and leukocytes; up to 100 µM heme), ex vivo in blood (30 µM heme), as well as upon intravenous (100 µmol/kg) or (35 µmol/kg) retroorbital injection in mice (in vivo) [40,41,42,43]. Subsequent steps on the molecular level that are explicitly induced by heme-driven TF upregulation were so far not analyzed.

Regarding the common pathway (Reactome, R-HSA-140875), the two networks share the following proteins: APC, FV, thrombin, fibrin, and fibrinogen (Figure 3C). The inhibitory effect of heme on APC has been described above. Whether heme prevents APC-mediated inactivation of the two cofactors, FV and FVIII, from both the intrinsic and the common pathway, has not yet been explored. In contrast, it is already known that heme can abolish the prothrombinase ([FXa + FVa])-catalyzed reaction, which was attributed to direct inhibition of the involved cofactor FVa in vivo (4 mg/kg heme injection in an AIP patient) [44]. The central enzyme of the common pathway, i.e., thrombin, has been suggested to be affected by strong heme binding in vitro as well, but the earlier reported decrease in its amidolytic activity in the presence of heme could not be reproduced in a recent study [36,45]. Although FXIIIa, the fibrin stabilizing factor, has been disproven as a heme-regulated protein [36], it is well known that fibrinogen itself binds heme in vitro, which results in fibrinogen binding to platelets as well as crosslinking and polymer formation in plasma and/or blood samples (up to 500 µM heme applied), ultimately leading to clot formation [46,47,48].

As such, direct heme binding has only been described for FVIII(a), fibrinogen, and APC so far, all possessing heme-binding affinities in the nano- to micromolar range (Figure 3D) [36,37,48]. In addition, several components of the blood coagulation cascade, which are present in the three databases, were not found in the herein established HemeThrombKG, e.g., FX(a), FX(a), and several coagulation inhibitors. Therefore, a specific screening for literature reports of these proteins in the context of heme signaling was conducted by entering the respective queries in PubMed, as this knowledge might not be covered in HemeThrombKG. However, no further relations were identified, proving the quality of the new knowledge graph and revealing novel targets for studying the effect of heme on the blood coagulation cascade (Figure 3D).

#### 3.2.2. Crosstalk of Heme within the Pathways of Platelet Activation

The crosstalk of heme with intracellular signaling pathways of the blood coagulation system was analyzed by superimposition of HemeKG 2.0 with the membrane-associated and intracellular proteins involved in the selected pathways from the databases KEGG [28], Reactome [29], and WikiPathways [30], revealing two receptors (Glycoprotein VI and thrombomodulin), one integrin (αIIbβ3), and eight intracellular proteins as common nodes (Figure 4A). The greatest overlap occurred between the HemeKG 2.0 network and a part of the platelet activation signaling pathways as extracted from KEGG (HSA04611, [28]; Figure 4B).

Initiation of platelet adhesion and activation is mediated either by adhesion proteins of the extracellular matrix (e.g., collagen and VWF) through interaction with integrins, glycoproteins on the platelet surface and/or by stimulation of receptors through agonists (e.g., ADP and thromboxane A2) [49,50,51]. As a result of intracellular signaling, the cytosolic level of Ca^2+^ ions increases and finally induces the change in platelet shape, secretion of granule content, and activation of the integrin αIIbβ3, which enables “communication” with plasma proteins, such as fibrinogen [51]. This, in turn, potentiates platelet adhesion, activation, aggregation, and spreading with the result of clot formation and retraction [51].

Superimposition of the HemeKG 2.0 network and the underlying pathways of these platelet activation mechanisms revealed that the two proteins of the extracellular matrix, collagen and VWF, as well as the glycoprotein VI, the integrin, αIIbβ3 and the intracellular proteins spleen-associated tyrosine kinase (Syk), AKT serine/threonine kinase (Akt), nitric oxide synthase (NOS), phospholipase C γ2 (PLCγ2), and protein kinase C (PKC) were common (Figure 4B). In contrast, several other effector proteins, which were found in the respective pathways in the KEGG database (part of HSA04611; [28]), were not found in HemeKG 2.0 (e.g., glycoprotein V, cGMP-dependent protein kinase or protein kinase G, or the extracellular-signal regulated kinase (ERK); Figure 4B). Thus, specific literature screening for reports of these effector proteins in the context of heme signaling was performed in the same way as described for the plasma proteins above (see Section 3.2.1). 

As a result, Fc receptor γ (FcRγ), phosphoinositide 3 kinase (PI3K), myosin, cSrc, and ERK were additionally found and highlighted in the network as well (Figure 4B; Table S1).

In the initial phase of heme-induced platelet activation, collagen and VWF seem to play a crucial role (Figure 4B). As already described herein (Section 3.2.1), heme excess (up to 100 µM tested) causes elevated VWF expression and string formation in vitro and in vivo, which might coordinate platelet adhesion to the vessel wall [20,23]. In an ex vivo model (mouse aorta), heme (1 mM)-triggered endothelial collagen expression was associated with increased platelet aggregation [52]. Until now, however, only the relevance of these proteins for platelet adhesion to the extracellular matrix and platelet aggregation caused by heme-driven upregulation of their expression has been described. Our analysis revealed that there is an indication for their contribution to heme-triggered platelet activation pathways as well (Figure 4B). In case of the pathway that follows platelet adhesion to collagen, its counterpart protein, GPVI, has been already described to bind heme with a rather low affinity (K_D_ ~29.4 µM), which induces platelet aggregation upon heme exposure in vitro (up to ~115 µM) [53]. The participation of FcRγ, which is intracellularly associated with GPVI, has also been suggested to participate in this process [54]. Usually, GPVI crosslinking or platelet adhesion to collagen induces phosphorylation of FcRγ, leading to recruitment and activation of intracellular signaling proteins, such as Syk, PI3K, and PLCγ2 [50,53]. Since heme seems to be capable of inducing platelet activation by targeting both collagen and GPVI, this might account for a direct and indirect activation of this pathway (Figure 4B) and, thus, an amplification of the observed effects. Syk and PLCγ2 phosphorylation in platelets was associated with heme (up to 50–115 µM) exposure as well [53,55]. This has been attributed to GPVI and C-type lectin-like receptor 2 (CLEC2) activation by heme [53,55]. CLEC2 itself is found in HemeThrombKG in relation to heme as well but did not occur as a common node in the crosstalk analysis since it is not yet curated into the database platelet activation signaling pathways.

In contrast to Syk and PLCγ2, the intermediary effector protein PI3K did neither occur in HemeThrombKG nor in HemeKG 2.0, because it has not yet been found in the context of heme and coagulation processes. Literature screening revealed that PI3K was identified as a mediator of heme-induced PLC phosphorylation in neutrophils (up to 30 µM heme tested) [56], which may apply for heme-driven intracellular platelet signaling as well. Activated PLCγ2 usually catalyzes phosphatidylinositol-4,5-bisphosphate (PIP_2_) hydrolysis to inositol trisphosphate (IP_3_) and diacylglycerol (DAG), leading to calcium mobilization and protein kinase C (PKC) activation [51]. A heme-induced increase in IP3 and DAG levels has not yet been described; however, elevated intracellular calcium levels and mobilization in platelets as well as PKC activation in neutrophils has already been monitored (in the presence of up to 20 µM heme) [46,57,58]. In general, calcium mobilization enables contractile activity through myosin, which leads to the characteristic shape change of activated platelets [50]. The calcium–myosin axis has not been highlighted in the context of heme signaling, but heme-induced oxidation of myosin in human skeletal muscle fiber segments and platelet shape change has been shown in vitro (up to 300 µM heme applied) [46,59]. Several of the other involved effector proteins (e.g., IP_3_ receptor, myosin light chain kinase, and RAS guanyl releasing protein 1) were not recognized and/or reported in the context of heme signaling (Figure 4B) and, thus, display suitable targets for future investigation of the underlying pathways of heme-triggered platelet activation.

Under conditions of heme excess, platelet aggregation through fibrinogen binding to platelets was observed (~11 µM heme applied) [46], proposing a heme-induced αIIbβ3-fibrinogen “communication”, since both components evolved in our crosstalk analysis as common nodes (Figure 4B). Furthermore, this integrin is capable of intracellular signaling induction. This, in turn, promotes platelet spreading, which also occurred as a node in HemeThrombKG. From the involved effector proteins, only the tyrosine kinase Src was already described to be activated by heme, but only in epithelial cells so far and not in platelets [60]. Src, in turn, can phosphorylate the FCγ receptor IIa (FCγRIIa) and induce Syk signaling (Figure 4B) [51]. This indicates that this pathway may be affected by heme as well, although FCγRIIa has not yet been reported in the context of heme signaling.

Apart from PLC activation (see above), PI3K can catalyze Akt phosphorylation, explaining its activation in platelets upon incubation with heme (2.5 µM) [57]. Heme-triggered platelet activation along with Akt phosphorylation was demonstrated to be dependent on TLR4 [57]. Although the TLR4 signaling pathway is highly pronounced in the HemeThrombKG network, it did not emerge during crosstalk analysis, since it is not yet recognized for its contribution to procoagulant processes in the databases. This pathway has been previously highlighted in the context of heme-driven inflammation and suitable future targets from this signaling were already pointed out [17].

Akt phosphorylation can further lead to eNOS activation in platelets, a hemoprotein, whose production has been reported to be influenced by heme [61,62]. Many of the subsequent effector proteins of this pathway have not yet been described in relation to heme signaling, but ERK has been found to contribute to heme signaling in other cell types (e.g., neutrophils) (Table S1) [43,57,63]. Furthermore, an interrelation of heme with the messenger molecules cyclic guanosine monophosphate (cGMP; in vivo), arachidonic acid, and thromboxane A2 (in vitro) in platelets has already been reported [46,64,65,66], suggesting the induction and progression of the respective pathways in the presence of heme.

In contrast to the collagen/GPVI-mediated platelet activation signaling, the pathway induced by platelet adhesion to VWF is largely unexplored as a potential route for heme signaling. None of the proteins of the GPIb-IX-V complex were mentioned in context with heme-mediated platelet activation. However, the pathway shares several effector proteins with the collagen/GPVI-initiated signaling, which could be activated by heme as part of the VWF-associated signaling pathway as well.

## 4. Discussion

The high prevalence of thrombosis in hemolytic disorders and the associated harmful complications emphasize the importance of the in-depth investigation of the molecular basis of heme-driven prothrombotic effects.

Thus, a mechanistic model of heme signaling in the context of blood coagulation is presented herein. “HemeThrombKG” represents the contextualization of the current knowledge about the interference of heme in the blood coagulation system. Furthermore, the analysis of the complex interrelations by enrichment and superimposition with information available from databases provides novel insights into underlying pathways with a focus on the enzymatic coagulation cascade and platelet activation signaling. In addition, crucial knowledge gaps were identified and highlighted that need to be targeted in future research.

In the past, several components of the blood coagulation cascade were reported to be affected by heme with contradictory outcome (anticoagulant (e.g., FV and FVIII) vs. procoagulant (e.g., APC and fibrinogen) signaling) but to date only three proteins (i.e., APC, fibrinogen, and FVIII(a)) out of this complex network were shown to bind heme in vitro. The heme-binding affinities along with the plasma levels of the coagulation factors (Figure 3D) could enable a temporal and heme concentration-dependent ranking of the processes in the blood coagulation system. The highest heme-binding affinity of the so far known heme-binding coagulation proteins exhibit FVIII (12.7 nM)/FVIIIa (1.9 nM) and APC (~400 nM), whereas fibrinogen possesses a moderate heme-binding affinity (~3.3 µM) [36,37,47]. However, while FVIII/FVIIIa and APC occur only in very low amounts in the plasma (subnano- to nanomolar range), fibrinogen is the clotting factor with the highest plasma concentration (micromolar range). Thus, it is highly probable that fibrinogen will be in the first line affected by heme, whereas APC and FVIII might only be regulated under conditions of heme excess. Still, several participating proteins have not yet been analyzed for these heme-binding characteristics, which were outlined in this study (e.g., FX, FXI, and various coagulation inhibitors). These should be analyzed for their potential heme-binding capacity in the future to enable a complete understanding of the temporal and spatial hierarchy of heme-triggered effects in the blood coagulation cascade and, thus, evaluation of the progression of hemolysis-driven thrombotic complications.

Beyond the effects on the coagulation cascade, heme has been associated with cellular events, including platelet adhesion, activation, and aggregation [46,53,55,57,67]. However, the underlying intracellular signaling pathways and, in particular, the interrelations of the already described effector proteins, are largely unexplored. To enable a more comprehensive analysis of potential intracellular signaling pathways, HemeKG 2.0, a combination of the previous (HemeKG [17]) and the novel (HemeThrombKG) heme knowledge graph, were used for the analysis of cellular signaling pathways. Thereby, the platelet activation signaling pathways were highly pronounced, emphasizing the importance of the collagen/GPVI signaling route for heme-driven platelet activation. As pointed out in this study, several effector proteins of this signaling pathway (e.g., Btk, SLP76, IP_3_R, MLCK, RASGRP, and Rap1) have not yet been described in the context of heme biology and should thus be experimentally investigated as potential heme-induced signaling proteins. The same applies for the VWF/GPIb-XI-V signaling route, where only VWF and messenger molecules were reported to be influenced under conditions of heme excess. However, it should be noted that a few of the herein included effector proteins (e.g., PLCγ2) have not yet been reported to be activated and/or induced by heme in platelets but only in other cell types, such as endothelial cells and leukocytes, which thus requires future experimental investigation in platelets. Beside the different cell types, the different heme concentrations that were used for the studies impede a direct comparison of the results. Furthermore, our analysis emphasizes the need for suitable and comparable in vivo studies that support the in vitro results and observations.

Beyond the herein described components of the blood coagulation system, other proteins occurred in the HemeThrombKG network that participate in the prothrombotic reactions, including receptors (TLR4, CLEC2 and thrombomodulin), adhesion proteins (selectin E, selectin P, ICAM1, and VCAM1), and several intracellular proteins (e.g., MAPK1, NLRP3, GGT1, and actin). Future analysis should include these proteins to generate a more complete picture of the procoagulant effects of heme.

The high complexity of the actions of heme as a modulator in the blood coagulation system is further evident by indirectly triggered prothrombotic mechanisms, such as LDL oxidation by heme or heme-released iron followed by endothelial cell damage [68]. These links were not analyzed in the present study but are already (at least partially) included in HemeKG 2.0 and are, thus, also available for further exploration.

Finally, HemeThrombKG as well as the combined HemeKG 2.0 were curated with standard vocabularies (e.g., from ChEBI [69] and MeSH [70]) using BEL, which makes it linkable to public databases. The networks and analyses performed on these networks have been made available at https://github.com/HemeThrombKG/HemeThrombKG, to allow the public to interactively explore the knowledge graphs and gain additional mechanistic insights [71]. Thus, researchers can easily inspect further relations and dependencies in the herein provided networks on their own. As such, the networks can be used to predict suitable drugs and their response in hemolytic disorders (e.g., SCD and PNH) in the future, supporting the selection of suitable drug candidates for the targeted treatment of hemolysis-associated thrombosis.

## 5. Conclusions

In conclusion, this study emphasizes the importance and relevance of the blood coagulation cascade and platelet activation signaling pathways for the reported prothrombotic effects of heme as occurring in hemolytic disorders. Furthermore, several effector proteins are highlighted for future studies, which will allow for a more detailed characterization of the pathophysiological outcome on the molecular level and, thus, establishment of novel perspectives for targeted treatment options of prothrombotic complications in patients with hemolytic disorders.

## Data Availability

The herein established knowledge graph (HemeThrombKG) and its inclusion into HemeKG (HemeKG 2.0) are publicly accessible at https://github.com/HemeThrombKG/HemeThrombKG.

## Supplementary Materials

The following supporting information can be downloaded at: https://www.mdpi.com/article/10.3390/jcm11195975/s1, Figure S1: Overview of the extracted knowledge from the common pathway databases; Table S1: Evidence for heme relations in the platelet activation signaling pathways from additional literature screening.
