# Supplementary material for: Exploring the Complex Network of Heme-Triggered Effects on the Blood Coagulation System
Source: J Clin Med. 2022 Oct 10;11(19):5975. doi: 10.3390/jcm11195975 (PMC9572022; doi:10.3390/jcm11195975)
Supplement: Supplementary file 1 [file jcm-11-05975-s001.zip › jcm-1954038-supplementary.pdf]

Supplementary figure

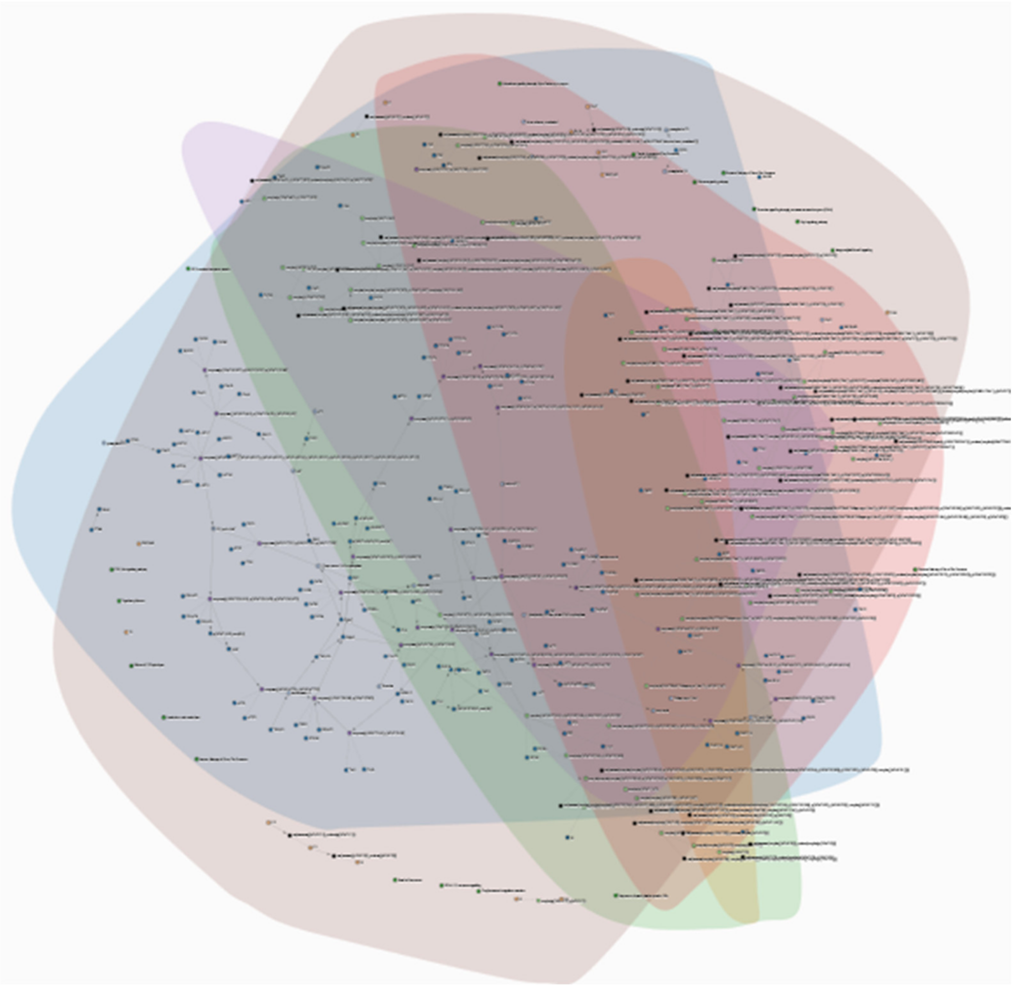

Figure S1. Overview of the extracted knowledge from the common pathway databases, involving pathways of platelet activation and of the coagulation cascade. The following pathways are included: HSA04611 (blue, “platelet activation”, KEGG), R-HAS-140834 (orange, “extrinsic pathway of fibrin clot formation”, Reactome), R-HSA-140837 (green, “intrinsic pathway of fibrin clot formation”, Reactome), R-HSA-140875 (red, “common pathway of fibrin clot formation”, Reactome), R-HSA-76009 (purple, “platelet aggregation/plug formation”, Reactome) and WP272 (brown, “blood clotting cascade”, WikiPathways).

Supplementary table

Table S1. Evidence for heme relations in the platelet activation signaling pathways from additional literature screening.

Relation	Evidence	PMID
"AA" and "heme"	Interaction of **arachidonic acid** and **heme** iron in the synthesis of prostaglandins.	6770594
"Src" and "heme"	We found that **heme** indeed affects the phosphorylation of key tyrosine residues in Jak2 and **Src**, and is therefore able to modulate Jak2 and Src activity.	21036157
"ERK" and "heme"	The ratio of phospho-**ERK** : ERK total was weakly modified by the injection of **heme** in WT mice (…).<br>(…) **heme** induces Akt phosphorylation and **ERK**-2 nuclear translocation in neutrophils.	33314778,<br>15265937
"FcRγ" and "heme"	**Heme**, a novel endogenous ligand shared by CLEC-2 and GPVI/**FcRγ**, activates human and murine platelets.	33843987
"myosin" and "heme"	Oxidation of sulfhydryl (SH) groups was detected in structural proteins (e.g., nebulin, α-actinin, meromyosin 2) and in contractile proteins (e.g., **myosin** heavy chain and myosin-binding protein C) as well as in titin in the presence of 300 μM **heme**.	33142923
"PI3K" and "heme"	Inhibition of ERK and **PI3K** pathways abolished **heme**-protective effects upon human neutrophils, suggesting the involvement of the Ras/Raf/MAPK and PI3K pathway on this effect.	15265937
